# Reviews of Books

**Published:** 1923-09

**Authors:** 


### REVIEWS OF BOOKS.

### THE HOUSE CONVENIENT.

**The House We Ought to Live In.** By John Gloag and Leslie Mansfield, F.R.I.B.A. (Duckworth; 7s. 6d. net.)

Mr. Gloag's previous books on furniture and decoration were admirable, and in this successor, in which he has had the help of an architect, he has completed a thoroughly useful trilogy. The collaborators begin at the beginning and lay the utmost stress upon the importance of planning and arrangement—one half of the inconveniences from which the householder suffers are the result of bad planning. We are glad to miss the usual tirade against the open fire. The authors recognise, as we all do, that it is wasteful and dirty, but it would be as easy to rob the average Englishman of his trousers as of his open fire, and doors and windows must be arranged in relation to it. Dust is so efficient a disease-carrier that books of this kind, which emphasise the importance of avoiding dust-traps, are valuable helps to sanitation. "The House We Ought to Live In" might profitably be read by manufacturers, to whom we must look for some of the improvements here suggested, such as a durable, easily cleaned, and silent material for stairs. But when the authors tell us that our bookcases ought to have glass doors we cannot avoid suspecting them of a certain leaven of wickedness. The amount of dust harboured by books is exaggerated; moreover, dust does them less harm than absence of air. Glass-fronted bookcases, too, are expensive, and the real book-lover is usually poor rather than otherwise. Also it would seem that the heartlessness of putting books in jail has not occurred to these severely practical writers.

We can almost forgive them for this heresy in gratitude for their attack upon the illusory water "trap" in the kitchen sink and elsewhere. They advocate, instead, a straight pipe from the sink waste, bent round easily at the bottom and allowed to discharge over an open channel having a properly constructed trap gully fitted with a little wire basket to catch scraps of refuse and prevent the drain from getting stopped. Yet this simple solution of the difficulty of preventing polluted air from ascending from the trap which fails to arrest it cannot be adopted because of the by-law compelling the use of the trap. In these, as in most other matters, the world learns wisdom slowly. The authors are not much more enamoured of the "labour-saving house" than of the trap in the scullery sink. To elaborate and complicated mechanical contrivances which will cook the dinner and wash the baby they prefer greater simplicity in arrangement, decoration and equipment—directions in which nothing can easily get out of order, and in which dust and inconvenience are greatly reduced. Simplicity, indeed, is their watchword, and they do their best to supply it.

The book contains many plans and designs for small houses and their equipment which are capable of teaching much to architects and their clients. No detail is beneath the authors, and they even give us a page of simple designs for the names of suburban houses. Their kitchen plans are especially excellent, and they add an appendix of useful heights and measurements, together with a glossary of technical terms. But the description of a corbel as "a member projecting from a wall to provide support for a beam" is not very illuminating—the unlearned would at once ask what a "member" is? The English language suggests that the expression should be "a projection." Also the English language requires that a fender-like arrangement in front of a fire should be called a "kerb," not a "curb," as the authors vainly imagine. Such defects detract hardly at all from the merits of an excellent book, the value of which is greatly enhanced by Mr. A. B. Read's drawings, which are at once clear and pictorial and properly and architecturally "hard."

### NURSING IN ENGLAND AND AMERICA.

**The British Nurse in Peace and War.** By Elizabeth S. Haldane, Ch., LL.D. (John Murray: 7s. 6d. net.)

**Nursing and Nursing Education in the United States.** (New York: The Macmillan Company: $2.00.)

Miss Haldane describes her book as a history of "a great series of achievements," and alludes to the nursing profession as having "a future practically unlimited." This, the considered opinion of one who has, by her intimate association with modern developments of the work, and her skill in the organisation of branches of the Services during the war, so built up for herself a reputation of sympathetic insight into the difficulties of other people, may well be of value to all thoughtful readers. The book will therefore appeal strongly to nurses who are always interested in knowing how their work—the oldest occupation and one of the newest professions for women—impresses one who, while standing outside its gates, is yet so closely knit to its aims and interests.

The seven chapters of Part I. trace the history of nursing from the days of Hippocrates through many changes and chances of the centuries during war and peace until its rapid development during the last sixty years to a great and important profession. Brief but interesting accounts are given of the origin and progress of the work as carried on in some of the larger hospitals, and the improvements accomplished during the nineteenth century, originating in the initiative and marvellous executive capacity of Florence Nightingale, whose death occurred in August, 1910, and not in 1900 as stated on page 134. The more modern movements of State registration, the organisation of special branches of nursing, including those connected with public health work, and the establishment of the College of Nursing, with the many improved facilities for the higher education of nurses, are also dealt with. For much of her information in this part of the book Miss Haldane has necessarily been indebted to the work of previous writers, but in the later chapters where she warms to the subject of the nurse in war, the personal note of experience and reminiscence makes them of special interest. Fresh developments in nursing, she reminds us, have always followed any great war. Indeed, war has had a direct and strong effect on the calling, and the two cannot be really separated. A trained and educated civil service was established as the result of the Crimean War. The Italian War of 1859 begat the Red Cross Society through Henri Dunant, and great developments took place through the Franco-German War of 1870. After the South African War of 1899-1902 Queen Alexandra's Imperial Military Nursing Service was instituted, followed by the establishment of Queen Alexandra's Royal Naval Nursing Service in 1907. Finally, the Royal Air Force Nursing Service came into existence in 1918.

The work done by these services during the recent War is well summarised by one who has used her opportunities well while sitting on hospital boards, and knows intimately the great achievements of nurses throughout those fateful years. Naturally, the work of the British Army Nursing Services in France holds the first place, but due recognition is also given to the valuable services rendered by the overseas nursing services from Canada, Australia, New Zealand, South Africa and the United States. In conclusion, the author appeals to all those who desire to show their gratitude for such services to "think out methods of making a great profession greater still." Nurse-readers may be reminded that in their own hands largely lies the power of doing this. Real improvement and development, whether of character or of a profession, must come from within.

According to statistics taken from the census of 1920 there are about 300,000 persons, male and female, described as "nurses" in the United States, half of whom are trained and registered, which gives roughly one trained nurse to about 700 of the population as a whole. The education and working conditions of so large a body of people are therefore of great importance to the welfare of the country, and "Nursing and Nursing Education" contains exhaustive reports on these matters as they exist to-day in the United States. The reports, which are the outcome of a conference on public-health nursing held at New York in December, 1918, are the work of a committee which was then appointed for the purpose. They are carefully compiled and do credit to the intimate and expert knowledge, also the thoroughness, of the investigators.

The book has two main divisions, the first dealing with the functions, the second with the training of the nurse, the latter occupying about two-thirds of the whole volume. Under the first heading are grouped the many ramifications of nurses' functions in the numerous departments of public health, also of those engaged in private nursing, and in institutions. Under the second, appear details of the student-nurses' career, the facilities offered for theoretical and practical training by the different institutions visited, questions of affiliation and post-graduate courses; the domestic, scholastic, social and professional aspects of hospital life are also all fully discussed. The field to be covered is wide, and the conclusions reached by the committee are, as a whole, well considered and sound, and the book should be useful to hospital administrators. Whatever differences of opinion may exist as to methods, the fact that the difficulties met with in the United States bear so striking a resemblance to those experienced in this country, cannot fail in its appeal to the sympathies of the British nursing world. Health visitors will find the description of an expert's day spent with a health-visiting nurse on her rounds both illuminating and amusing.

### ORGANISATION OF TUBERCULOSIS WORK.

**The Tuberculosis Worker.** By Phillip P. Jacobs, Ph.D. (Williams and Wilkins Co., Baltimore, U.S.A. $3.50.)

This book really does "fill a long-felt want"—fills it very well, too. It is general knowledge that the tuberculosis death-rate is falling more rapidly in America than in almost every other country in the world, and Dr. Jacobs gives many useful clues to the superiority of the Americans in this matter. Apparently the tuberculosis campaign has been organized in America with the same thoroughness and efficiency as the motor-car industry and other great undertakings—nothing is left to chance.

The primary aim of the book is the discussion of technique—to bring to public health workers a knowledge of the experience gained during the past twenty years in this field. The tuberculosis worker is urged to see himself as a salesman, selling health. Much useful advice is given with regard to dealing with the Press. The tuberculosis worker who wishes to be successful must pull the wires leading to editorial offices with infinite skill and tact, and the author has come to regard editors as nice but human. Editors do not like to have "editorials" thrust upon them, "but most editors are constantly looking for suggestions for editorials, just as ministers are looking for suggestions for sermons. The enterprising tuberculosis executive will be able to secure editorial comment by giving suggestions to the editor from time to time." Useful hints, moreover, are provided with regard to lecturers; the ideal lecturer is one who is so full of his speech that it fairly oozes out of him without a halt or incessant references to notes. He must also have enthusiasm and vitality and be able to adapt himself to different audiences.

The qualifications of a "tuberculosis secretary" are surely a little exacting. He should be "an administrator, a promoter, a dreamer, a student, a good fellow and a diplomat." The author not only gives useful advice, but he also tells much about the past work of the American National Tuberculosis Association. Its activities are numerous, and we need only mention one to show how thorough and systematic the American way is. It has undertaken to track down and expose "fake" cures and questionable agencies. There are more than 500 "fake" consumption cures and an increasing number of questionable agencies trading upon the tuberculosis campaign, and whenever it is possible the Association endeavours by legal or other methods to destroy these mischievous and parasitic agencies. The 314 pages of this unusual book are crammed with hints and advice of infinite value both to the physician and the layman willing to take part in the tuberculosis campaign.

